# The effect of changing stool collection processes on compliance in nationwide organized screening using a fecal occult blood test (FOBT) in Korea: study protocol for a randomized controlled trial

**DOI:** 10.1186/1745-6215-15-461

**Published:** 2014-11-26

**Authors:** Hye Young Shin, Mina Suh, Hyung Won Baik, Kui Son Choi, Boyoung Park, Jae Kwan Jun, Chan Wha Lee, Jae Hwan Oh, You Kyoung Lee, Dong Soo Han, Do-Hoon Lee

**Affiliations:** National Cancer Control Institute, National Cancer Center, 323 Ilsan-ro, Ilsandong-gu, Goyang, Gyeonggi-do 410-769 South Korea; Department of Health Education and Management, Health Science College, Ewha Womans University, 52, Ewhayeodae-gil, Seodaemun-gu, Seoul, 120-750 South Korea; Center for Cancer Prevention & Detection, National Cancer Center Hospital, National Cancer Center, 323 Ilsan-ro, Ilsandong-gu, Goyang, Gyeonggi-do 410-769 South Korea; Center for Colorectal Cancer, National Cancer Center Hospital, National Cancer Center, 323 Ilsan-ro, Ilsandong-gu, Goyang, Gyeonggi-do 410-769 South Korea; Department of Laboratory Medicine and Genetics, Soonchunhyang University Bucheon Hospital and Soonchunhyang University College of Medicine, 170, Jomaru-ro, Wonmi-gu, Bucheon-si, Gyeonggi-do 420-767 South Korea; Department of Gastroenterology, Hanyang University Guri Hospital, 153, Gyeongchun-ro, Guri, Gyeonggi-do 471-701 South Korea; Department of Laboratory Medicine, Center for Diagnostic Oncology, National Cancer Center Hospital, National Cancer Center, 323 Ilsan-ro, Ilsandong-gu, Goyang, Gyeonggi-do 410-769 South Korea

**Keywords:** Colorectal cancer, Colorectal neoplasm, Early detection of cancer, Fecal occult blood test, Randomized control trial, Factorial design

## Abstract

**Background:**

Colorectal cancer (CRC) screening by fecal occult blood test (FOBT) significantly reduces CRC mortality, and compliance rates directly influence the efficacy of this screening method. The aim of this study is to investigate whether stool collection strategies affect compliance with the FOBT.

**Methods/Design:**

In total, 3,596 study participants aged between 50 and 74 years will be recruited. The study will be conducted using a randomized controlled trial, with a 2 × 2 factorial design resulting in four groups. The first factor is the method of stool-collection device distribution (mailing vs. visiting the clinic) and the second is the type of stool-collection device (sampling kit vs. conventional container). Participants will be randomly assigned to one of four groups: (1) sampling kit received by mail; (2) conventional container received by mail; (3) sampling kit received at the clinic; (4) conventional container received at the clinic (control group). The primary outcome will be the FOBT compliance rate; satisfaction and intention to be rescreened in the next screening round will also be evaluated. The rates of positive FOBT results and detection of advanced adenomas or cancers through colonoscopies will also be compared between the two collection containers.

**Discussion:**

Identifying a method of FOBT that yields high compliance rates will be a key determinant of the success of CRC screening. The findings of this study will provide reliable information for health policy makers to develop evidence-based strategies for a high compliance rate.

**Trial registration:**

CRIS:KCT0000803

Date of registration in primary registry: 9 January, 2013.

**Electronic supplementary material:**

The online version of this article (doi:10.1186/1745-6215-15-461) contains supplementary material, which is available to authorized users.

## Background

Colorectal cancer (CRC) is a major health care concern worldwide, with a high incidence and mortality rate. About 1.23 million new cases of CRC and 608,000 associated deaths were estimated worldwide in 2008[1]. In developed western countries, CRC mortality and incidence are decreasing, while the incidence remains high in Asian countries such as Japan, China, South Korea, and Singapore[2, 3]. In Korea, CRC caused 25,782 new cases (35.9 per 100,000) and 7,645 (10.4 per 100,000) deaths in 2010[4].

Based on reports that CRC screening by a fecal occult blood test (FOBT) significantly reduces CRC mortality[5–8], several countries have implemented FOBT screening at the national or regional level[9]. In England, the National Health Service Bowel Cancer Screening Program (NHSBCSP) adopted a biennial guaiac-based FOBT for those aged 60 to 75[10]. In Korea, the National Cancer Screening Program (NCSP) offers annual fecal immunochemical testing (FIT) to people aged 50 years or older[11].

Although a FOBT is relatively simple, inexpensive, and noninvasive compared to colonoscopy, the rate of FOBT screening varies from 20% to 71% among countries[9]. In Korea in 2012, the rate of FOBT screening was only 25.7%[12]. Several barriers, including health system, provider, and patient factors, contribute to the low rate of FOBT screening[13–16]. In Korea, the system-related barriers to an efficient screening process have been reported to be the requirement for two consecutive clinic visits to receive the stool device and submit the sample, and the use of a stool container that has a short, thick sampling probe to collect stool samples[17]. To overcome these barriers, two interventions are being considered. The first is to distribute stool-collection devices by mail, which requires only one clinic visit to submit the sample. The second is to provide a stool sampling kit consisting of a small test tube including a longer, thinner sampling probe that is easier to poke into the stool to collect stool specimens. Through these interventions, this study will determine the most effective strategy to increase compliance and satisfaction in FOBT screening. This determination is based on the assumption that changing the stool-collection process (especially as regards the type of stool-collection device) will not affect the results of FOBTs, but may affect compliance and satisfaction. We will therefore compare the rates of positive FOBT results between the two stool devices to validate our assumptions.

## Methods

### Trial design

**Figure 1:**
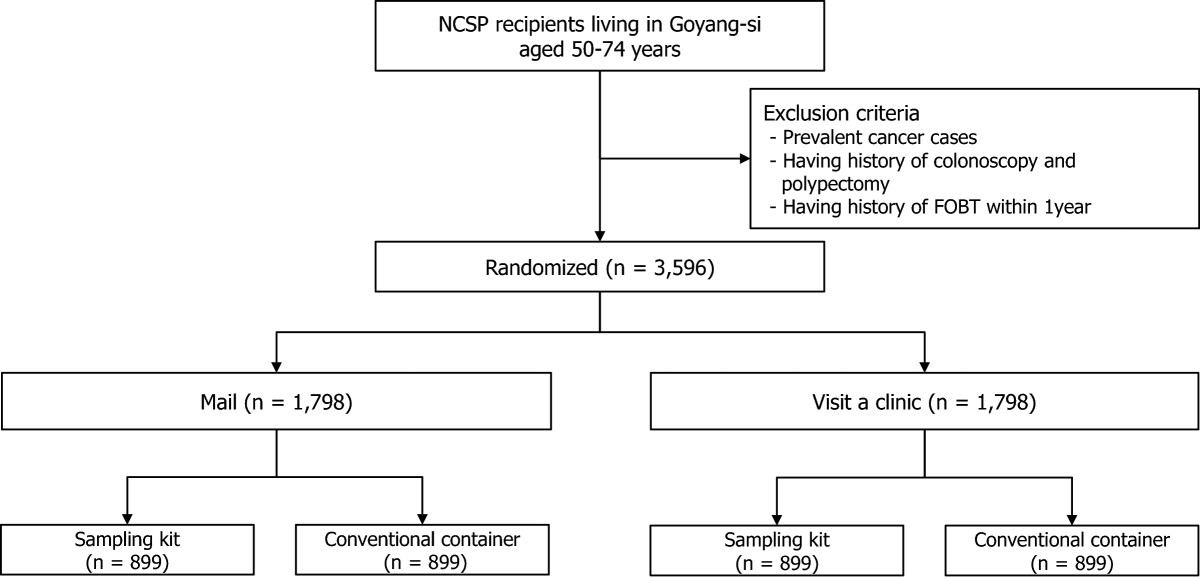
**Flow chart of the study.**

The study will be conducted using a 2 × 2 factorial randomized control trial (RCT) design resulting in four groups. The first factor is the two methods of distributing stool devices (mailing vs. visiting a clinic), and the other is the two stool devices (sampling kit vs. conventional container). Thus, the study will be composed of four groups from two factors (allocation ratio1:1:1:1): (1) sampling kit received by mail; (2) conventional container received by mail; (3) sampling kit received at the clinic; (4) conventional container received at the clinic (control group) (Figure 1).

### Participants

Individuals aged 50 to 74 years living in Goyang will be recruited from February 2013 to February 2015. The National Cancer Center (NCC) in Goyang has sufficient resources to screen all NCSP recipients living in the Goyang urban area. The study will take place at the NCC Center for Cancer Prevention and Detection. Invitation letters including a brief description of FOBT screening and the purpose of the study will be mailed to target participants, and those who want to participate in the study will provide verbal consent via telephone. After study staff confirms that they meet the study criteria, eligible participants will be randomly allocated to one of four groups. Written informed consent will be obtained from all participants upon visiting the clinic to submit stool specimens within two weeks after receiving a stool device. Exclusion criteria include: a prior diagnosis with any cancer including colorectal cancer; a history of colonoscopy or polypectomy; or a history of a FOBT more recent than one year. Ethical approval has been obtained from the Institutional Review Board at the NCC (NCCNCS-12-683).

### Interventions

**Figure 2:**
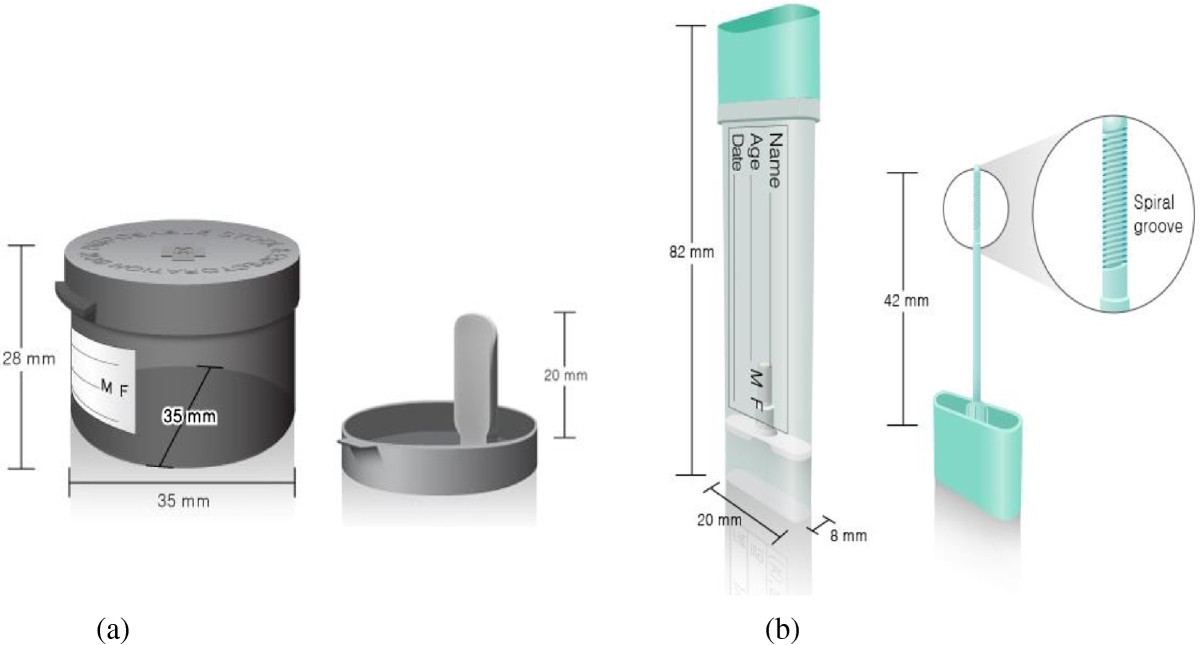
**Illustrations of stool devices: (a) conventional container; (b) sampling kit.**

There will be four groups: sampling kit received by mail, conventional container received by mail, sampling kit received at the clinic, and conventional container received at the clinic (control group). Sampling kits are being purchased from Eiken Chemical Co., Ltd., Tokyo, Japan. Compared to the currently used conventional container, the new sampling kit is thinner and smaller, with a thin (4.2 cm long) sampling probe attached to the cap that allows easier insertion into the stool. The tip of the sampling probe has a spiral groove to collect stool specimens (Figure 2). The sampling probe with the collected stool (approximately 10 mg) may then be reinserted into the kit. These stool specimens are analyzed in an OC-SENSOR DIANA machine (Eiken Chemical Co., Ltd., Tokyo, Japan) designed for quantitative immunochemical FOBTs and the sampling kits fit this equipment. Stool specimens in conventional containers are transferred to sampling kits for immunochemical FOBTs at the laboratory because the conventional containers do not fit the analyzer. Mailed sampling kits or conventional containers will include an instruction leaflet. All participants who receive stool devices will be instructed in methods of stool collection via telephone or face-to-face by research staff.

### Outcome measures

The primary outcome will be compliance with FOBT screening; compliance will be defined as the proportion of individual participants who submit stool specimens to the National Cancer Center within two weeks following receipt of collection devices. The secondary outcome will be participant satisfaction and intention to be rescreened in the next screening round as assessed by questionnaire at the clinic. The questionnaire will use a five-point Likert scale (1 = strongly disagree, 2 = disagree, 3 = uncertain, 4 = agree, 5 = strongly agree) with three dimensions: satisfaction with each process, from receiving stool devices to submission of specimens at the clinic; overall satisfaction with the entire process; and intention to be rescreened. The tertiary outcome will be the rates of positive FOBT results using sampling kits vs. conventional containers. Positive results are defined as those exceeding 100 ng/mL blood. All participants who undergo FOBT will be informed of the screening results via mail within three weeks, and those whose results are positive will be recommended to undergo colonoscopy. Colonoscopy or further biopsy for abnormal lesions will be performed by highly qualified colonoscopists upon provision of written consent at the NCC. Histopathological results will allow comparison of the detection rates of advanced adenoma or cancer between sampling kits and conventional containers. All outcomes of the study will be explained to respondents upon interview and prior to obtaining written informed consent.

### Sample size calculation

Study participants will be divided into two groups according to the method of receiving the stool-collection device. To determine the sample sizes of two groups, we will establish the null hypothesis (H_0_) and alternative hypothesis (H_1_) using the difference *p*_*1*_*-p*_*2*_, where *p*_*1*_ is the expected compliance rate in the group receiving collection devices by mail and *p*_*2*_ is the expected compliance rate in those receiving them at a clinic.

$$\begin{array}{l} H_{0}:p_{1}=\;p_{2}\left(\delta =p_{1}-\;p_{2}=0\right)\;\text{vs}.\\ H_{1}:p_{1}>p_{2}\left(\delta =p_{1}-\;p_{2}>0\right) \end{array}$$

The expected compliance rate of participants visiting a clinic is based on that observed in 2010 by the NCSP, which was 30.8%, and the compliance rate of participants receiving stool devices by mail is estimated at 36% based on the assumption that the rate will be at least 5% greater.

$$N=\frac{{\left\{Z_{\frac{\alpha }{2}}\sqrt{\left(r+1\right)\bar{p}\left(1-\bar{p}\right)}+Z_{\beta }\sqrt{rp_{1}\left(1-p_{1}\right)+p_{2}\left(1-p_{2}\right)}\right\}}^{2}}{r{\left(p_{1}-p_{2}\right)}^{2}}$$

Based on the above formula, 1,398 people will be needed in each group to detect the difference *p*_*1*_*-p*_*2*_ in the proportions using a two-tailed test and 80% power (1-β) at a 5% significance level. However, continuity correction tests will be needed to estimate very closely approximate values, and thereby a sample size of 1,438 in each group will be required. Finally, to allow for a 20% loss to follow-up, we will enroll an additional 350 participants per group, resulting in a final total for each group of 1,798 participants; thus, a total of 3,596 participants will be enrolled in the study.

### Randomization

After providing verbal consent by telephone, those who are eligible will be allocated to one of four groups by a computer-generated randomization program using the SAS 9.2 statistical software (SAS Institute, Inc., Cary, NC, USA). The sequence will be generated by simple randomization. Participants will be subjected to different trial conditions depending on the number they are allocated.

### Statistical analysis

Compliance rates for FOBT screening will be compared among the four groups by a chi-square test. The satisfaction and intent to rescreen using a FOBT will be assessed by analysis of variance (ANOVA). The rates of positive FOBTs and the rates of advanced adenoma or cancer detected by colonoscopy using the different stool devices will also be analyzed by a chi-square test. The adjusted odds ratios (ORs) and 95% confidence intervals (CIs) of each outcome will be calculated using multiple logistic regression. All data will be analyzed using the SAS software, version 9.2 (SAS Institute, Inc., Cary, NC, USA).

### Study integrity

This study will use a randomized study design to evaluate whether distribution using mail delivery and an improved stool-collection device increase FOBT screening compliance. Participants will be recruited through letters of invitation and randomly assigned to one of four groups (three interventions and one control group). Participants will be included only if they submit a stool sample to the NCC in person within two weeks after receiving a stool device, and these participants will be rewarded with small gifts. To maintain consistency in data collection, we will assign two well-trained research staff members to oversee data management, supervise all steps, and train assistant staff throughout the study duration.

### Data management

All information collected will be covered by the Personal Information Protection Act. Participants’ personal information such as name, identification number, and contact number will be not used until they have signed a consent form. Research staff will manage all information to ensure confidentiality under the personal data regulations. Participants’ identifiable data will be retained only until the end of the study. Any information will be eliminated upon participant request.

## Discussion

All Koreans aged 50 years or older are screened for CRC using a FOBT by the National Cancer Screening Program (NCSP). The success of this program relies on the rate of compliance, which has not been satisfactory. Likely factors contributing to low compliance include the time-consuming nature of the stool-collection process and its inconvenience. Therefore, this study will evaluate two modifications to the stool-collection process to increase compliance, which relies on two assumptions. First, the time savings and convenience of the mailing intervention increase the accessibility of FOBT screening. Second, the sampling kit reduces the inconvenience and embarrassment involved in stool sampling.

A potential limitation of this study is that it is limited to an urban area. The NCSP covers all of Korea, whereas we will carry out the study in one city, with a population of almost 1 million. This population may not reflect the characteristics of those residing in rural areas or small cities, who are in greater need of screening and have less access to health care. However, mailing of stool-collection devices has been shown to be effective in increasing compliance in FOBT screening[18], and may have an even greater effect in rural areas or small cities.

This study will be the first RCT in collaboration with CRC screening in the NCSP. A randomized design is required to determine the effectiveness of novel interventions and to establish evidence-based strategies. Utilizing the NCSP’s population and resources will enable generalization of the study outcomes to the Korean population. The cost-effectiveness of CRC screening, FOBT compliance and detection rates of adenoma or cancer will also be estimated for the general population. Finally, this study will provide information for policy makers to develop evidence-based strategies for use across the health care system. Furthermore, application of the Consolidated Standards of Reporting Trials (CONSORT), which require that checklists and flow diagrams be included in publications of trial results[19], will provide more reliable and valid evidence to health policy decision makers.

## Trial status

The trial started in February 2013.

## Authors’ original submitted files for images

Below are the links to the authors’ original submitted files for images. Authors’ original file for figure 1 Authors’ original file for figure 2

## Abbreviations

ANOVA: analysis of variance
CI: confidence interval
CONSORT: Consolidated Standards of Reporting Trials
CRC: colorectal cancer
FIT: fecal immunochemical test
FOBT: fecal occult blood test
NCC: National Cancer Center
NCSP: National Cancer Screening Program
NHSBCSP: National Health Service Bowel Cancer Screening Program
OR: odds ratio
RCT: randomized controlled trial.
