# Supplementary figures and images for: The effect of changing stool collection processes on compliance in nationwide organized screening using a fecal occult blood test (FOBT) in Korea: study protocol for a randomized controlled trial
Source: Trials. 2014 Nov 26;15:461. doi: 10.1186/1745-6215-15-461 (PMC4289376; doi:10.1186/1745-6215-15-461)

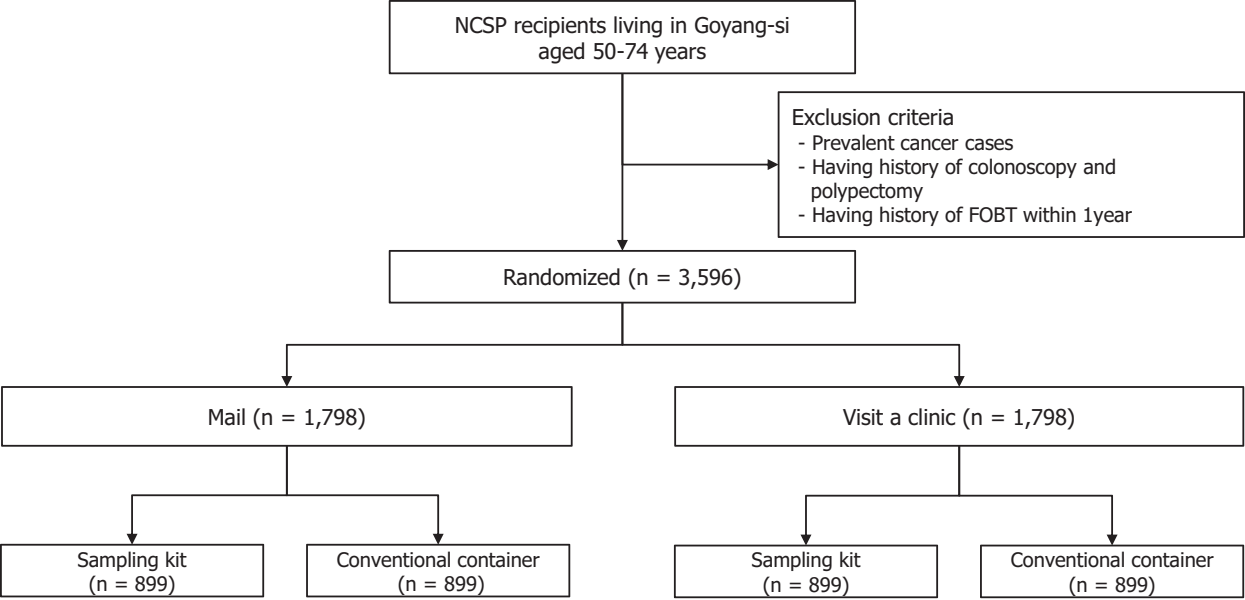

Supplement: Supplementary file 1 — Authors’ original file for figure 1 [file 13063_2014_2356_MOESM1_ESM.pdf]

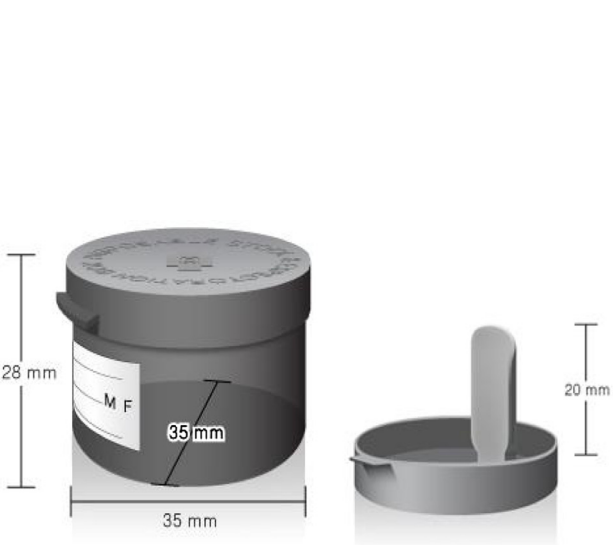

(a)

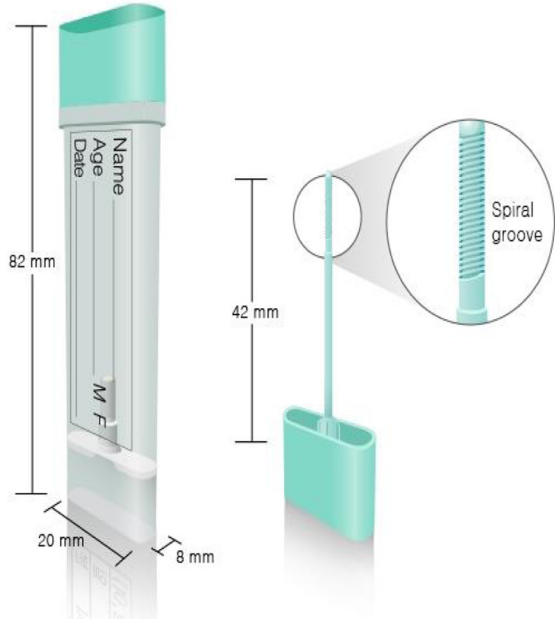

(b)

Supplement: Supplementary file 2 — Authors’ original file for figure 2 [file 13063_2014_2356_MOESM2_ESM.pdf]
